# Innovative rehabilitation intervention in a young stroke patient with Parinaud syndrome: A case report

**DOI:** 10.5339/qmj.2025.30

**Published:** 2025-03-08

**Authors:** Thajus Asirvatham, Ajay Boppana, Premraj Issac Chandran, Muhaiadeen Sheik Abubacker Jamaludeen

**Affiliations:** ^1^Department of Occupational Therapy, Qatar Rehabilitation Institute, Hamad Medical Corporation, Doha, Qatar; ^2^Qatar Rehabilitation Institute, Hamad Medical Corporation, Doha, Qatar *Correspondence: Thajus Asirvatham. Email: TAsirvatham@hamad.qa

**Keywords:** Parinaud syndrome, stroke, rehabilitation, function

## Abstract

**Background:**

Parinaud syndrome is a rare condition that can arise as a consequence of strokes, hemorrhages, and neoplasms. Due to the debilitating after-effects of this condition, such as functional dependency, a high risk of falls, decreased mobility, and increased caregiver burden, immediate and holistic intervention is essential.

**Case presentation:**

A 40-year-old male patient presented with Parinaud syndrome following an episode of stroke. Physical examination on admission revealed self-care dependence, limited mobility and upper limb function, limited visual fields, and reduced therapy tolerance.

**Discussion:**

The patient was treated with belay glasses, an innovative strategy, to assess functional changes. This intervention resulted in notable improvements in overall function, contributing to greater independence in daily activities. Standardized assessments indicated improvements in self -care, mobility, and hand function. The use of belay glasses not only increased visual function but also facilitated overall functional gain.

**Conclusion:**

Parinaud syndrome is an unusual condition that can arise following a stroke or brain trauma. This case report aimed to explore the functional gain of a patient diagnosed with Parinaud syndrome during an 8-week rehabilitation program, using a multidisciplinary approach and the application of prism glasses. Clinically significant differences were observed using belay glasses, representing an innovative intervention strategy.

## Introduction

Visual and visuo-perceptual problems are common after a stroke. The role and intervention related to these visual deficits are limited by decreased knowledge, lack of expertise, and time-consuming evaluations, ultimately hampering the rehabilitation process.^1^ Such visual disturbances can jeopardize patient safety and impede the ability to perform essential activities such as reading, navigating, and moving around, as well as locating and grasping objects.^2^ In addition, visual deficits may significantly increase the risk of falls and adversely affect overall quality of life.^3^ Therefore, it is imperative to implement significant measures to address the impairments and establish appropriate rehabilitation goals. Parinaud syndrome is a rare condition classically characterized by a triad of symptoms: impaired upward gaze, convergence retraction, nystagmus, and pupillary hyporeflexia. Patients may present with difficulty looking upward, blurred near vision, diplopia, and associated neurological symptoms.^4^ The primary focus of management is to treat the underlying cause before irreversible changes occur.^5^ The role of rehabilitation through a holistic approach in managing such a condition is still under investigation. The most common causes of Parinaud syndrome include tumors and midbrain infarctions, with 65% of cases arising after hemorrhagic strokes, despite its relatively low incidence and prevalence.^6^ Previous research has also indicated that the occurrence of Parinaud syndrome following a stroke is a rare and unusual complication, as illustrated by a case report documenting the syndrome following a transient ischemic attack.^7^ Although the range of rehabilitation strategies available to facilitate recovery is limited, it has been proposed that the use of prism glasses may offer significant benefits in enhancing functional outcomes. One such study indicates that the use of prism glasses improves arm function, hemineglect, and activities of daily living (ADL) among stroke patients, emphasizing their potential for futuristic and broader application in rehabilitation.^8^ The belay glasses, which incorporate prismatic lenses, are primarily designed for rock and mountain climbing to enable users to avoid looking upward while navigating trails, thereby reducing neck strain and preventing falls. These glasses work by the principle of reflecting light from above into the observer's eyes, thereby expanding peripheral visual awareness.^9^ However, there is currently a lack of research evidence supporting the use of belay glasses in the context of stroke rehabilitation. Prismatic lenses, such as Fresnel prism glasses, have been used to aid in the recovery and functional improvement of stroke patients.^10^ The aim of this study was to explore the potential of belay glasses in assessing their effectiveness in correcting Parinaud syndrome following a stroke. The article presents the case of a patient who was managed in a tertiary care rehabilitation hospital in Qatar. The patient provided signed informed consent for the publication of the case report and accompanying images. Additionally, ethical approval was also obtained from the Medical Research Centre at the Hamad Medical Corporation (MRC-04-23-673).

## Case Presentation

**Figure 1.**
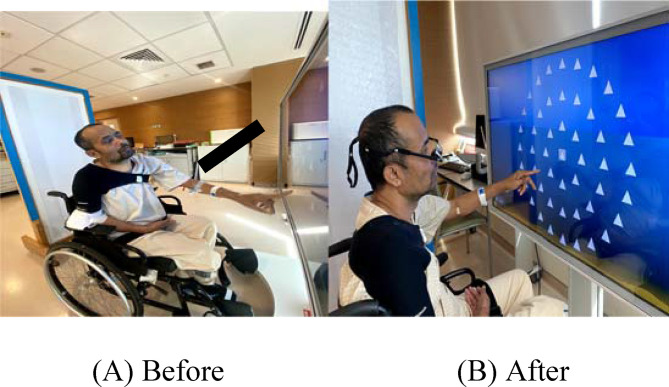
Comparison of conditions before and after the use of belay glasses during Retouch (cognitive session). (A) The patient exhibiting upward gaze palsy, resulting in an awkward sitting posture with the head thrown backward. (B) The patient demonstrating visual scanning on a Retouch screen while wearing belay glasses, highlighting a more comfortable sitting posture with the head maintained in a neutral position.

A 40-year-old male patient, diagnosed with a stroke, presented to the emergency department with clinical signs of Parinaud syndrome and a new onset of right-sided weakness. Subsequently, he was admitted to the intensive care unit for one week, where he was acutely managed and treated until medically stable. Following this, the patient was shifted to the neurology ward for further examination and interdisciplinary rehabilitation management. Repeated computed tomography of the head and magnetic resonance imaging were conducted every four weeks for the next two months, revealing a large left ganglio-capsular bleed with mass effect involving the left corona radiata and left thalamus, as well as multiple chronic microbleeds, with no evidence of intracranial vascular anomalies. The patient exhibited impaired upward gaze, which led to restrictions in participation due to vertical gaze limitations and dependence on assistance for self-care activities, attributed to limited visual acuity. This condition resulted in compensatory movements such as leaning the head backward to accommodate for the impaired visual field (Figure 1(A)). Additionally, mild right facial weakness and dense right hemiplegia of both the upper and lower extremities were noted. The patient showed poor to fair sitting balance and was non-ambulant. The multidisciplinary plan included education regarding the current condition, an overview of the inpatient rehabilitation program, expected functional outcomes, and the length of stay, with a focus on enhancing ADL independence such as eating, grooming, bathing, dressing, and toileting. The plan was also to facilitate mobility independence and train independence in the bladder management program. At the community level, the plan included reviewing for aids, equipment, and recommendations for home modifications, all aimed at ensuring safe ambulation and reintegration into the community. The occupational therapy (OT) evaluation included an initial evaluation, followed by biweekly progress notes. Performance in the ADL and functional assessments were measured using the Functional Independence Measure (FIM), a tool that measures a patient's level of functional independence on a seven-point scale, both at baseline and at regular intervals.^11^ Physical examination revealed significant right-sided hemiplegia with complete paralysis of the upper and lower extremities. Hypertonicity was present in the shoulder flexors (grade 1), shoulder adductors (grade 1), elbow flexors (grade 1+), and wrist and long finger flexors (grade 1+), as measured by the modified Ashworth scale.^12^ Sensation was impaired in both the upper and lower extremities, with the right hand exhibiting no functional capability. The initial power of both the upper and lower extremities was graded 0/5, according to the Medical Research Council grading system. The functionality of the upper extremity was measured using the Action Research Arm Test (ARAT), which consists of a 19-item observational measure that assesses upper extremity performance in terms of coordination, dexterity, and functioning in stroke populations.^13^ Visual field testing was conducted periodically every two weeks at the hospital to monitor any changes in visual fields following sessions of visual perceptual retraining. Physical therapy (PT) evaluations were performed within the same time frames. The initial assessment was performed upon admission, followed by progress notes every two weeks and discharge notes. Belay glasses^14^ were used during therapy and while performing self-care activities to facilitate visual field expansion. The adherence to the intervention was closely monitored by both therapists and assigned nurses, except during self-care and sleeping times. Visual field testing was performed using virtual reality devices such as Retouch,^15^ in order to assess the effectiveness of the glasses and the rehabilitation process. The study included data extracted from electronic patient records, from which assessments and outcome measure scores were taken. The patient attended OT and PT sessions for one hour each day, five days a week, for 8 weeks, after which he was discharged home. No adverse effects were reported during the intervention.

From the time of admission to discharge, significant improvements in self-care scores were observed, increasing from 44 to 83 points on the FIM scale. Additionally, mobility status, as measured by the Functional Ambulation Category (FAC), improved from gradings 0/5 to 2/5. Minimal changes were noted in the ARAT, which assesses upper limb function, with scores increasing from 0 to 18 points (Figure(B)). Participation in therapy showed significant improvement, as evidenced by the extension of session length from 30 minutes to 120 minutes, with adequate rest intervals between the sessions. Visual field testing revealed improvements in the number of cues needed to perform the task. The patient reported an improvement in vision, an expanded visual field, and a reduced fear of falling. Furthermore, the patient expressed satisfaction with the belay glasses, attributing them to an enhanced quality of life and overall well-being.

## Discussion

A collaborative multidisciplinary team approach yielded positive functional gains for this patient. This is supported by previous literature that highlights enhancements in gait and complete independence in activities such as toileting and personal hygiene, emphasizing the importance of a multidisciplinary team approach to facilitate functional improvements.^16^ Prism glasses have shown benefits in the scope of rehabilitation by assisting patient awareness of their neglected side and correcting postural deficits.^17^ The application of prisms, exemplified by the belay glasses, has been instrumental in enhancing patient awareness of visual field restrictions and promoting changes in correcting postures while sitting in a wheelchair and during standing activities. These prism glasses serve as a compensatory strategy designed to facilitate visual field scanning and gaze stability.^18^ Prisms have been used in improving overall outcomes in stroke patients with visual problems by maximizing visual perceptions and helping individuals adapt their vision to their observable space.^19^ In the present case, the use of prisms contributed to the enhancement of visual functionality, thereby enabling the patient to perform daily activities with increased confidence and improved participation in therapy sessions. It is well noted that prisms or prism glasses are effective tools in the rehabilitation and intervention of peripheral visual deficits, homonymous hemianopia, and other refractive errors.^20^ There is limited research on the use of prisms in the rehabilitation of gaze palsies. This study represents one of the first investigations into the comprehensive use of prism glasses in a rare case of Parinaud syndrome, thereby providing a novel perspective on rehabilitation in such rare scenarios. Another study used a similar intervention of prism glasses to induce gaze shift, address strabismus, and correct abnormal head posture in a case of traumatic Parinaud syndrome. The prisms proved effective in correcting strabismic deviations and enabling gaze shifts within limits, thereby facilitating functional capabilities.^21^ This encouraged the authors to examine the potential benefits of belay glasses, which are also prismatic in nature, to determine whether they could yield positive functional outcomes. This case report is the first to explore the use of such specialized prisms to enhance functionality, which constitutes a significant strength of the study. Although the glasses may not solely account for the observed positive functional gain, they were used as an adjunct to facilitate recovery and enhance participation in various activities.

## Conclusion

The findings presented in this case report indicate a significant increase in functional gain regarding self-care independence, with minimal changes to upper limb function and therapy engagement. This emphasizes the importance of therapies, such as OT and PT, in facilitating further recovery and enhancing function. The application of belay glasses as an additional intervention strategy proved beneficial in facilitating overall functional gain and enhancing the patient's overall performance. Despite their relatively limited application in rehabilitation contexts, the positive outcomes obtained with belay glasses highlight their potential utility. To accelerate recovery and enable early discharge to home, it is essential to use innovative rehabilitation strategies, including the use of belay glasses in visual compensation and the rehabilitation of neurological disabilities characterized by impaired gaze palsies. In addition, a diverse array of interventions can be used to facilitate rehabilitation efforts and optimize functionality without resorting to invasive procedures. Future research should aim to explore the effectiveness of such innovative rehabilitation interventions with a larger sample size, yielding more profound insights.

### Acknowledgments

We express our heartfelt gratitude to the management of QRI, Hamad Medical Corporation for their unwavering support in the development of this case report. Their commitment to advancing healthcare research has been invaluable. We are thankful for their vision and encouragement.

### Competing interests

The authors have no conflicts of interest to declare.
